# Cell–Cell Mating Interactions: Overview and Potential of Single-Cell Force Spectroscopy

**DOI:** 10.3390/ijms23031110

**Published:** 2022-01-20

**Authors:** Peter N. Lipke, Jason M. Rauceo, Albertus Viljoen

**Affiliations:** 1 Biology Department, Brooklyn College of the City University of New York, 2900 Bedford Avenue, Brooklyn, NY 11210, USA; 2 Department of Sciences, John Jay College of the City University of New York, New York, NY 10019, USA; jrauceo@jjay.cuny.edu; 3 Louvain Institute of Biomolecular Science and Technology, UCLouvain, Croix du Sud, 4–5, bte L7.07.07, 1348 Louvain-la-Neuve, Belgium

**Keywords:** atomic force microscopy, cell–cell mating, adhesion, single-cell force spectroscopy, yeasts, bacteria, conjugation, gametes

## Abstract

It is an understatement that mating and DNA transfer are key events for living organisms. Among the traits needed to facilitate mating, cell adhesion between gametes is a universal requirement. Thus, there should be specific properties for the adhesion proteins involved in mating. Biochemical and biophysical studies have revealed structural information about mating adhesins, as well as their specificities and affinities, leading to some ideas about these specialized adhesion proteins. Recently, single-cell force spectroscopy (SCFS) has added important findings. In SCFS, mating cells are brought into contact in an atomic force microscope (AFM), and the adhesive forces are monitored through the course of mating. The results have shown some remarkable characteristics of mating adhesins and add knowledge about the design and evolution of mating adhesins.

## 1. Introduction

There is a growing interest in the adhesive interactions occurring between cell pairs that allow for mating to occur [1,2,3]. In prokaryotic cells and yeasts, the role played by surface adhesion proteins (adhesins) in selective interactions has been the subject of intense research for many years. These interactions play key roles in colonization, biofilm formation, and, in pathogens, direct interactions with host tissues and cells that lead to infection [4]. Moreover, the role of force in these interactions is gaining recognition [5,6,7]. While measures of affinity under equilibrium conditions are very useful in the characterization of receptor–ligand interactions, adhesive interactions often occur under out-of-equilibrium conditions, that is to say that binding needs to occur and be strong under physical stress. The study of adhesive receptor–ligand interactions, thus, requires appropriately adapted approaches. Accordingly, great progress has been made in atomic force microscopy (AFM) force spectroscopy approaches [8]. Herein, we briefly review current findings on the adhesive interactions that play a role in cell–cell mating. We then make a case for the use of AFM force spectroscopy methodologies in this burgeoning field by highlighting recent advances made in the more general field of bacterial and fungal adhesion, as well as in the field of intercellular DNA transfer. Lastly, we discuss specific ways in which AFM force spectroscopy methods can assist future investigations and characterizations of the cell–cell adhesive factors in mating.

## 2. Mating Systems: Short Overview

In general, mating systems are highly complex, and they depend on the regulation of complex gene expression networks to regulate both temporal and spatial coincidence of interacting gene products. Nevertheless, in all organisms, the process requires two characteristics: specificity of the interaction and a long enough contact time to ensure cellular fusion and/or gene transfer. Because mating systems are both complex and fast-evolving [9,10,11,12,13,14], there are a wide variety of strategies to achieve these characteristics.

Gametic recognition depends on the interaction of mating adhesins. Through combinations of strong affinity and low dissociation rates, these adhesins must be species-specific and must maintain the binding interaction long enough for subsequent steps in mating to occur. The slow *k*_off_ characteristics are due to the combination of affinity and avidity. Affinity is the pairwise strength of binding, i.e., the ratio of dissociation rate to association rate between monovalent receptor and ligand. Such interactions typically give equilibrium dissociation constants in the range of 10^−5^–10^−9^ M. For interactions in the weaker range (K_D_ ≈ 10^−5^–10^−7^ M), avidity is key. Avidity is the increase in binding and the lowered chances of dissociation when adhesins and ligands are clustered. Avidity increases exponentially with the number of adhesive bonds. When there are two sets of adhesin–ligand bonds holding two cells together, the cells are only dissociated at the product of the individual adhesion *k*_off_ rates. For instance, if *k*_off_ = 10^−2^ s^−1^, that implies ~70 s as a half time for dissociation. If there are two sets of adhesive bonds, then the cellular dissociation rate will be the product of the individual *k*_off_, i.e., 10^−4^ s^−1^ or half-time of 5000 s (>1 h). Thus, multiple adhesive bonds between the gametes can compensate for low affinity between adhesin and ligand.

### 2.1. Issues in Species Specificity of Mating

**Figure 1:**
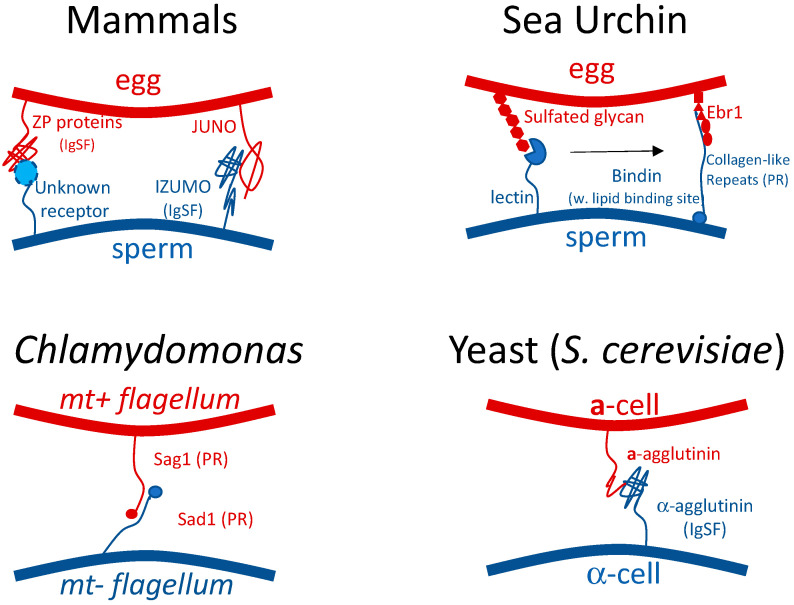
Cartoon models of some eukaryotic mating adhesins, as described in the text. Protein with Greek key β-sandwiches (IgSF) and Pro-rich repeats (PR) as recurring motifs.

In eukaryotes, successful mating requires specific recognition and binding between gametes, followed by complex interactions that result in dissolution or penetration of cell gametic surface layers, and finally cell fusion. There are complex signaling pathways that can initiate gametic chemotaxis, recognition, dissolution of the gametic glycocalyx, and finally initiation of membrane fusion. There is no taxon in which all the steps are well characterized. In the context of conjugant recognition and binding, relatively few pairs of interacting adhesins are known, and only in yeast do we also know detailed biochemical characteristics. The best characterized mating systems are in sea urchin, *Chlamydomonas*, baker’s yeast, and bacteria. Even in these systems, we know little about the initial adhesion events in gamete recognition (the limited examples that have been studied are illustrated in Figure 1).

#### 2.1.1. Fertilization in Metazoa

In mammalian fertilization, the zona pellucida proteins and the Izumo1/Juno pairs are known to be essential for binding and fertilization [13]. The egg protein ZP2 is a zona pellucida protein required for species-specific sperm recognition, and sperm binding triggers polyspermy blocking activities. The ZP2 ligand has not yet been identified, but there is species specificity in binding. The ZP proteins share a common ‘Greek key’ β-sheet sandwich fold with gamete recognition proteins from sea urchin, abalone, and yeast [1,12,14,15,16]. The mammalian sperm protein Izumo1 and the egg protein Juno are required components for fertilization [14,15]. These proteins interact at the level of the oolemma (egg membrane). The interaction is dependent on a low-affinity/high-avidity interaction. The half-time for dissociation is only around 0.5 s; hence, each interacting pair of proteins is unstable. High-avidity patches would need to have 10’s to 100’s of interacting pairs to maintain the interaction over longer times. There is some specificity, but it is not absolute, because hamster Juno binds Izumo1 from several mammalian species. In contrast human Juno binds human Izumo1, but not mouse Izumo1 [14,15]. The Juno–Izumo1 binding is under intense research as a possible target for contraceptive design.

#### 2.1.2. Sea Urchins

Fertilization in sea urchins (echinoderms) is well studied; nonetheless, few binding partners have been identified. Unidentified sperm lectins initially bind species-specific sulfated glycans in the egg jelly coat, and then trigger the acrosome reaction [17,18,19]. Bindin is an acrosomal protein of sperm that can agglutinate conspecific eggs, but not heterospecific eggs [20]. Bindin-deleted sperm are infertile. Bindin is localized in granules; thus, it is presumably highly avid. Ligands for Bindin include phosphatidyl ethanolamine and two egg proteins: Ebr1 and a 350 kDa glycoprotein. The latter protein inhibits fertilization at µM concentrations and egg aggregation at sub-µM concentrations [21]. We do not know about affinity or kinetics of interactions with its various ligands, but a K_D_ in the range of 10^−6^ M to 10^−8^ M might be a first guess.

#### 2.1.3. Green Algae

In the green alga *Chlamydomonas*, complementary adhesins Sag1 in mt+ cells and Sad1 in mt− cells initiate species-specific contact leading to gametic fusion [22]. The initial adhesion is at the tips of the flagella, but the adhesins relocate more basally and eventually to the polar cell fusion site. The interaction is strong and may indeed be irreversible, since the Sag1–Sad1 complex is proteolytically shed into the medium [23].

#### 2.1.4. Yeasts

In baker’s yeast, *Saccharomyces cerevisiae*, two haploid mating types *MATα* and *MAT***a** can bind to each other and mate by fusion to form a diploid zygote. As in metazoa, many genes are involved in mating, and many processes must be completed for successful mating to occur. The steps include pheromone signaling, specific adhesion, establishment of cell polarity, cell-wall fusion and dissolution, membrane fusion, and nuclear fusion [24].

Facile genetics has resulted in the identification of many components of the *S. cerevisiae* mating system including complementary sexual adhesins [25,26]. α-Agglutinin is expressed on *MATα* cells, and its expression is increased following exposure to the sex pheromone a-factor secreted by the opposite mating type *MAT***a**. α-Agglutinin is homologous to the human fungal pathogen *Candida albicans* agglutinin-like adhesins (Als) and, like them, has two Greek-fold, Ig-like domains and a stalk that covalently anchors the binding domains to the cell-wall glucan. α-Agglutinin binds with high affinity to its ligand **a**-agglutinin expressed on the surface of *MAT***a** cells. **a**-Agglutinin has two subunits, one which anchors it to the cell wall, and a 69-residue glycopeptide that contains the binding determinants, especially the C-terminal sequence. **a**-Agglutinin is highly upregulated following exposure of the *MAT***a** cells to the pheromone α-factor secreted by *MATα* cells; therefore, there is a positive feedback loop that increases the surface concentration of the adhesins [1,25,26].

The sexual adhesins are necessary for efficient mating in liquid media but are dispensable for mating on solid surfaces [27]. Extensive in vitro analyses with soluble forms of the agglutinins show a tight interaction (K_D_ ~ 10^−9^ M) with an extremely slow dissociation rate with *k*_off_ ~10^−4^ s^−1^ [28]. In fact, dilution of α-agglutinin/**a**-agglutinin leads to dissociation of about half the complexes in 2 h, comparable to the time necessary for completion of mating. Binding is accompanied by a temperature-dependent conformational shift that stabilizes the interaction. It is in these interactions that SCFS has now allowed us to see functional consequences of the biochemical characteristics.

#### 2.1.5. Bacterial Conjugation

Bacterial conjugation is well characterized, and most systems rely on type IV secretion systems (T4SS), a modified pilus in which a dozen or more gene products assemble to facilitate binding to recipient cells and gene transfer as a protein–DNA complex [29,30,31,32,33,34,35]. Nevertheless, relatively few interactions of the adhesins themselves with target cell ligands have been biophysically characterized. As in yeast, cell–cell adhesion is partly dispensable for mating on solid surfaces; however, in liquid media, loss of adhesins reduces conjugation frequency by many orders of magnitude [29,33]. Thus, in the bacteria, conjugation mechanisms are also diverse, but are analogous to fertilization; they include mechanisms for DNA mobilization, binding to recipient cells, and penetration of the cell wall and plasma membrane.

Adhesion is the major function of mating pair stabilization (MPS) [30], required to hold donor and recipient cells close enough for long enough to allow DNA transfer. Among the Firmicutes (including all Gram-positive bacteria), the adhesins appear not to be directly associated with the T4SS. Rather, these separate proteins are present on the outer layer of the wall and bind to ligands exposed on the outer layer of recipient cells. These adhesins are covalently integrated into the wall through Sortase-mediated anchorage to peptidoglycan, analogous to GPI anchors in fungal adhesins. Among MPS systems, the PrgB adhesin of *Enterococcus faecalis* binds to e-DNA and lipoteichoic acid, and it facilitates biofilm formation and mating [32,33].

The adhesion systems in eukaryotic and prokaryotic mating discussed under Section 2 are summarized in Table 1.

## 3. Characteristics of SCFS

**Figure 2:**
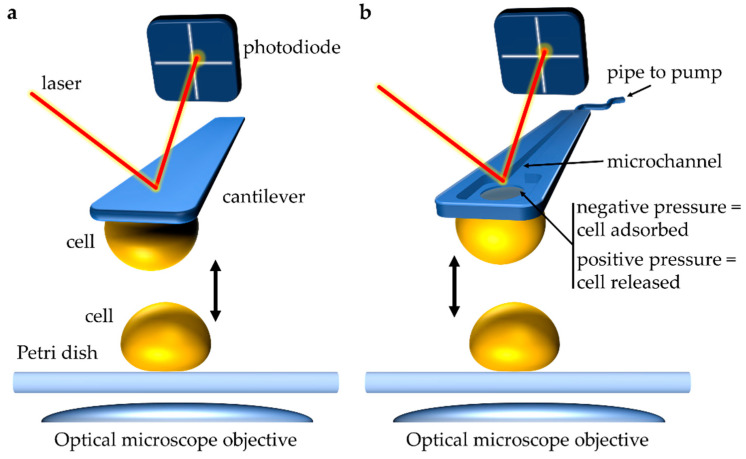
Atomic force microscopy (AFM) single-cell force spectroscopy (SCFS) to study cell–cell interactions during mating. (**a**) Conventional approach to performing single-cell SCFS experiments. A cell attached to a soft cantilever via (bio)chemical means (for example via poly-l-lysine coating) is brought into contact with a sample, like another cell. Interactive forces between the sample and the tip cause deflection of the cantilever. A laser beam focused on the cantilever and reflected onto a photodiode captures the deflection of the cantilever, which can be quantified as force (N). (**b**) In single-cell fluidic force microscopy (FluidFM) a micron-sized aperture connected to a microchannel within the cantilever is used to capture a cell through pressure differential. Connecting the cantilever through a microfluidics system to a pump allows controlling pressures at the apex. As such, the cantilever is approached toward a single cell, and a negative pressure is generated causing noninvasive adsorption of the cell to the cantilever. This cell probe can then be used to probe interactions with other single cells. Similarly, application of positive pressure can release the captured cell. This approach has aided in the study of yeast cell–cell interactions during mating.

Single-cell force spectroscopy (SCFS) has been instrumental in improving our understanding of cell–substrate adhesion, cell–cell adhesion, and cell–host adhesion down to the molecular level (for recent reviews, we direct the reader toward [6,8]). Briefly, the principle of SCFS consists of attaching a single cell to a soft, force-sensitive AFM cantilever (Figure 2a). This cell probe can then be used to measure interactions with other surfaces, including those of other single cells. By bringing the cell probe in contact with the surface of another cell and subsequently retracting it away, while recording the forces between the two cells, force–distance (FD) curves are generated, from which the strength of adhesion between the two cells can be determined accurately. Importantly, the velocity at which the cell probe is approached and pulled away from the sampled cell can also be controlled precisely. For most measurements, an arbitrary constant approach/retract velocity of ~1–10 µm·s^−1^ is used to roughly match physiological conditions [37]. However, varying the retraction velocity allows assessing the effect of different force loading rates on the tensile strength of molecular complexes formed between adhesive molecules on the surfaces of the interacting cells. Performing such dynamic force spectroscopy experiments can provide information on the energy landscape underlying forced unbinding of receptor–ligand complexes [38]. AFM can also be utilized to investigate the binding dynamics between single cells and polypeptide ligands. We previously used this technique (called single-molecule force spectroscopy, AFM-SMFS) to detect and analyze the unfolding of *C. albicans* Als5p. In those experiments, a single yeast cell expressing a V5 epitope-tagged Als5p was immobilized onto a polymer membrane. Als5p-V5 was detected, and the adhesion forces were measured using AFM tips coupled to anti-V5 antibodies [39]. Notably, recent AFM-SCFS experiments have revealed binding dynamics associated between the SARS-CoV-2 spike protein and human ACE2 receptor. In those experiments, the receptor-binding domain (RBD) of the SARS-CoV-2 spike protein was attached to the AFM tip, and the purified ACE2 receptor or ACE2-expressing cells were immobilized onto their respective surface [40,41].

A new, exciting AFM technology that has improved the ease, robustness, and speed of SCFS experiments is fluidic force microscopy (FluidFM), which relies on AFM cantilevers containing a microchannel that is connected to a pump [42]). These microfluidic probes are capable of extracting contents from or injecting foreign material into eukaryotic cells through precise pressure control at the aperture [43,44]. Alternatively, FluidFM allows capturing single cells in a fast, noninvasive manner that does not require (bio)chemical adhesives that may interfere with cellular function. Next, the captured cell is used to probe another surface such as a different cell or model substrate (Figure 2b) [43,44,45]. The reversibility of the adhesion between cell and probe makes it possible to pick up and release a single cell in a controlled manner, and this technique has been applied to isolate single bacteria expressing a desired phenotype from a mixed microbial population [46]. Regarding force spectroscopy, FluidFM has been applied to uncover the biophysical dynamics governing bacterial adhesion to hydrophobic surfaces [47] and to quantify forces in the adhesion to abiotic surfaces of yeast and mammalian cells [39].

### 3.1. SCFS Application to Studying Yeasts

#### 3.1.1. Intercellular Adhesion

In *C. albicans*, the Als cell wall glycoproteins mediate attachment to host surfaces. AFM force spectroscopy studies have provided critical insight into the biophysical mechanisms underlying Als-mediated adhesion to host ligands and other Als-expressing yeast cells. These studies have pioneered techniques for the study of cell–cell adhesion in mating. For example, sawtooth patterns in force extension curves observed in SMFS studies helped unravel how sequential unfolding of specific tandem repeat domains within Als adhesins conveys strength and resilience to the protein, which are required for its activity under physical stress [48]. SCFS studies have propelled our understanding of the cell–cell adhesive interactions when cells are subjugated to mechanical force. Specifically, conformational changes in Als adhesins [49] and *S. cerevisiae* flocculins [50] expose amyloid sequences that favor β-sheet interactions between the laterally arranged cell surface proteins, leading to the formation of adhesin-rich nanodomains that strengthen cell–cell binding. Both of these phenomena were reproduced in a more recent FluidFM study [51].

The binding forces between *C. albicans* and host immune cells have also been quantified by SCFS. The formation of multiple molecular bonds was demonstrated to occur rapidly between lectin receptors exposed on macrophage surfaces and mannan carbohydrates exposed on the *C. albicans* cells, thus offering an explanation for how macrophages engulf the pathogen quickly after initial contact [52]. The interaction with dendritic cells was also investigated, and the role of *N*-glycosylation in the pathogen recognition receptor DC-SIGN was found in cell stiffening during *C. albicans* binding [53].

**Figure 3:**
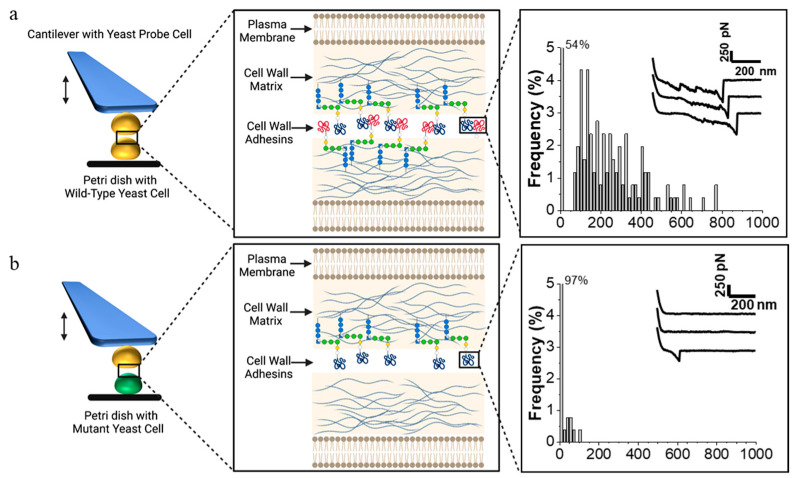
SCFS demonstration of the cellular role of a gene product in cell interaction. FluidFM-based SCFS allows for many different strains to be used as cell probes successively. Each cell probe can be brought into contact with many different cells in a petri dish. (**a**, **left**) A yeast cell probe attached to the cantilever interacts with a cell in the petri dish on the surface of the microscope stage through multiple cycles of contact and retraction of the probe. In the case of a mating experiment, the two cells are of opposite mating types. (**middle**) Details of cell surface showing cell membrane and cell wall, including blue glucan fibers and adhesins covalently crosslinked to the glucan through modified C-terminal GPI-anchors. (**right**) Histogram of rupture forces on successive adhesion events as the cells are brought into contact, and then separated; the inset shows force–distance curves for three representative contacts. (**b**) The situation when the cell on the microscope stage is a mutant cell, in this case, lacking a cell surface adhesin. This phenotype would result from mutation in the adhesin gene, in a gene required for cellular localization of the adhesin, or in a gene that regulates expression of these genes. In this case, the experiment yields infrequent cell–cell adhesions; thus, 97% of the F–D curves show no force needed for separation. The F–D plots are intended as illustrations only and are reprinted from Dehullu et al. [11].

While earlier studies of Als interactions relied on the use of purified binding partners (e.g., antibodies) attached to AFM tips, recent FluidFM-based SCFS analyses unraveled the forces in Als homophilic binding occurring between single pairs of yeast cells [51,54]. FluidFM-based SCFS allows monitoring of the interaction between two single living yeast cells as they are brought into contact and subsequently separated, while recording adhesin-dependent attractive forces existing between the cell pair as a function of distance or time (Figure 3a). Force curves may exhibit specific profiles characteristic of the unfolding patterns of adhesins under study (Figure 3a, right), while specificity may be further supported using appropriate negative controls, such as mutant cells not expressing the adhesin (Figure 3b).

#### 3.1.2. Intercellular Adhesion during Mating

As such, FluidFM-based SCFS was recently applied to study adhesive interactions in yeast mating by bringing a pair of *MATα* and *MAT***a** cells in contact while meticulously controlling contact force and time [36]. Subsequent pulling revealed the forced extension and unbinding behavior of single interacting α-agglutinin/**a**-agglutinin partners [36]. Under minimal contact time (0.1 s) and contact force (0.25 nN) conditions, a relatively weak single bond strength of approximately 100 pN was observed for pheromone-induced cells. Strikingly, increasing either the contact time (1 s) or the contact force (1 nN) led to sharply increased magnitudes of rupture forces, as well as the frequencies of binding events occurring. This supports the notion of high-avidity α-agglutinin/a-agglutinin interactions playing an important role in strengthening adhesion during *MATα–MAT***a** mating. A question was whether contact-facilitated agglutinin expression over longer timescales may result in enhanced adhesion between cell pairs. Meticulous control of cell–cell contact is one of the unique capabilities of SCFS. Therefore, this question could be addressed by taking force measurements before and after an extended cell–cell contact. In essence, the adhesion force was measured between a mating pair, and then the cells were brought into contact for 30 min, after which a set of adhesion force measurements were again performed. This approach revealed that initial *MATα–MAT***a** cell contact promotes surface expression of agglutinins, resulting in increased strength of the adhesive contact between a mating cell pair. AFM is uniquely suited to answer another question: how does mechanical stress (for example, caused by fluid shear flow encountered during the normal lifestyles of most microbes) influence the bond strength of the *MATα–MAT***a** cell pair? By increasing the pulling speed of the cell probe, the effect that high force loading rates have on forced unbinding of molecular complexes can be investigated. Remarkably, it was observed that high loading rates correlated with increased bond strength. This result indicates that mechanical stress may enhance agglutinin-dependent intercellular adhesion required for mating. This study proved the concept of using SCFS as a powerful tool to study cell–cell mating interactions, a major scientific breakthrough that can now be applied to various mating systems.

### 3.2. SCFS Application to Studying Bacteria

#### 3.2.1. Bacterial Adhesion

Recent SCFS-based analyses in bacteria demonstrated remarkable mechanostability of the single-molecular complexes formed by a number of MSCRAMM (microbial surface component-recognizing adhesive matrix molecules) adhesins and their ligands. The prototypical example is that of *Staphylococcus epidermidis* SdrG binding to the human plasma protein fibrinogen (Fg). X-ray crystallographic studies unraveled an exotic binding mechanism called “dock, lock, and latch” wherein a terminal peptide of Fg is tightly confined within a groove located at the center of the adhesin’s immunoglobulin-like head domain [55]. This observation fueled speculation that the complex may resist high tensile loads relevant to the in vivo context of an arterial infection. Direct evidence for this was brought by SCFS studies that quantified the mechanical strength of single SdrG–Fg interactions occurring on single living *S. epidermidis* cells, showing that tensile loads exceeding 2 nN could be sustained before rupture occurred [56,57]. To put it into perspective, this is more than ten times stronger than the streptavidin–biotin complex and practically as strong as a covalent bond [58]. Follow-up SCFS and SMFS studies found similar mechanostabilities for several related *Staphylococcal* MSCRAMM adhesins (reviewed in [59]). Another discovery that was made for several of these MSCRAMM adhesins is the enhancement of their binding to their ligands by mechanical stress; that is, the faster the complexes were pulled apart, the greater the tensile load that they could sustain [60,61]. These results exemplified the property of catch bonding, i.e., bonds whose stability (lifetime) is enhanced by tensile load [62,63]. In *S. pseudintermedius*, AFM force clamp mode analysis demonstrated that the complete interaction between the MSCRAMM adhesins SpsD and Fg is enhanced by tensile load up until a certain point where it transitions to a conventional slip bond (bond lifetime decreases with tensile load) behavior [64].

Another way in which SCFS has been useful in the study of bacterial adhesion is in the investigation of direct pathogen–host cell interactions. For example, SCFS studies with staphylococcal probes and endothelial cells revealed that the ternary complex consisting of fibronectin (Fn) binding protein A (FnBPA), Fn, and α5β1 integrins (exposed on the endothelial cells) is considerably stronger (~800 pN) than the Fn–integrin bond in isolation (~100 pN) [65]. This observation supports a hypothesis wherein FnBPA binding to Fn causes conformational changes in Fn such that cryptic integrin-binding sites are revealed and its binding to the integrin is allosterically enhanced. Similar observations were later made for a ternary complex involving *S. aureus* ClfA (an ortholog of SdrG), Fg, and the endothelial cell integrin αVβ3 [66].

#### 3.2.2. Bacterial Adhesion in the Context of Mating

**Figure 4:**
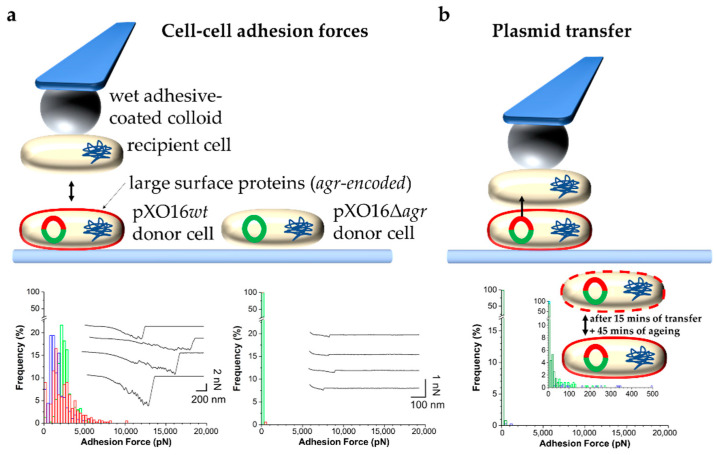
SCFS to probe and control bacterial conjugation. (**a**) Measuring forces in the adhesion of single pXO16*wt* or pXO16Δ*agr* donor cells to single recipient cells. Top: Schematic of the approach. The pXO16 plasmid is represented by a red and green (pXO16*wt*) or green (pXO16Δ*agr*) circle. The red part indicates the Agr region encoding large surface proteins (also indicated as a red outline around cells) that bind to recipient cells not expressing such proteins. Bottom: Histogram plots of the adhesion forces between recipient and donor cells carrying pXO16*wt* (**left**) pXO16Δ*agr* (**right**). (**b**) Controlling plasmid transfer. Top: Schematic showing the approach consisting of (i) contact between recipient and donor cells for 15 min allowing DNA transfer, (ii) followed by retraction and rest for 45 min allowing maturation, i.e., proper expression and surface localization of *agr*-encoded proteins, and (iii) eventual measurements of cell–cell adhesion forces to evaluate changes in cell surface properties caused by plasmid transfer. Bottom: Histogram plots of the adhesion forces showing a loss of strong adhesion as a result of the presence of *agr*-encoded proteins on both cells. Using the resulting pXO16*wt*-cell probe to probe a naïve recipient cell restored strong adhesion as shown in the bottom left panel of (**a**). Figure adapted with permission from [69].

*Bacillus thuringiensis* is the organism used in Bt insecticides and is, thus, relatively well-studied. The subspecies *israelensis* harbors a large plasmid (pXO16) that confers the ability to transfer itself to many other strains [67,68]. MPS is a result of the Agr^+^ adhesin encoded on the plasmid. Neither its structure nor its ligand is known. Here too, SCFS studies have brought novel insights into intercellular binding occurring during mating. An SCFS approach was devised where a single recipient cell was attached to a special bioadhesive-coated colloidal probe, which could be used to force probe a donor cell attached to a surface (Figure 4a). It was found that that the bonds induced between single donor and recipient cells are remarkably strong (~2 nN) and depended on the presence of a 27 kb fragment of the pX016 plasmid encoding two giant proteins containing collagen-binding B-type domains, as well as the peptidoglycan-anchoring LPXTG motif [69]. An exciting possibility brought by the technique was to mechanically induce the transfer of the plasmid between cell partners and to measure the time of plasmid transfer (Figure 4b). The assay consisted of bringing a recipient cell probe and donor cell into contact for 15 min allowing plasmid transfer. Afterward, the cells were separated, and the recipient cell was allowed to mature for 45 min during which Agr adhesins could be expressed and surface localized. When such a new Agr^+^ cell probe was used to probe the original Agr^+^ donor cell, a dramatic drop in adhesion forces and frequencies was observed. On the other hand, large adhesion forces and frequencies were observed when this cell probe was used to force probe recipient cells not expressing Agr adhesin. These results, thus, highlighted that Agr adhesins bind strongly to ligands that are masked in other donor cells by the adhesins themselves. To date, this *Bacillus* mating system is the first to have been investigated by AFM force spectroscopy techniques. Thus far, FluidFM approaches have not yet been applied to studying bacterial conjugation. Therefore, we envisage that the advantages of this approach, including its higher throughput and the reversibility of the attachment of cells to the probe, would be of immense value in the study of intercellular adhesion required for DNA transfer.

## 4. Summary and Conclusions

A limited number of adhesive factors important for the process of mating to occur have now been discovered and characterized in both eukaryotic and prokaryotic organisms (Table 1). Conventional biochemical approaches to studying receptor–ligand interactions have pointed to high avidity in these interactions where parallel bonding, sometimes involving clustering of binding partners on the surfaces of the cells, maximizes the strength of intercellular adhesion. As adhesive interactions often need to combat shear forces that pull interacting partners apart, their characterization requires techniques that allow quantifying the tensile forces that they can resist before they rupture. As we have detailed here, AFM SCFS approaches are suited to this task.

In the field of pathogen–host adhesion, specifically, SCFS approaches have been critical to several recent key discoveries. These include the discovery of the strongest non-covalent single-molecular interaction in nature existing between a bacterial adhesin and its ligand [56,57], that the binding strength of certain adhesins is enhanced by mechanical stress [60,61], and that some adhesins form unusual catch bonds, whose bond lifetime is enhanced by tensile load [64,70]. These studies serve as models for the cell–cell adhesions during mating.

In the field of mating, SCFS has now been used in a bacterial and a fungal mating system. Both studies have revealed strong interactions (nN per cell pair) due to multiple binding interactions between specific gene products. In the yeast system, we can compare the strength of cell–cell binding to thermodynamic and kinetic characteristics, as well as known cell surface concentrations. In yeasts, the cell–cell interactions are actuallystrengthened under tensile force. This technique can now assay enough cell pairs to allow phenotypic characterization of the interactions between cells with specific mutations or gene deletions in known or putative adhesins [36,69] or differences in surface expression levels [36]. What else lies ahead? Other possibilities offered by AFM force spectroscopy remain to be tapped into; for example, dynamic force spectroscopy can be applied to quantify out-of-equilibrium thermodynamic and kinetic parameters of single-molecular interactions as has been demonstrated for single virus–cell interactions by the Alsteens group [71,72,73,74,75,76,77,78]. This methodology can serve as an excellent platform to investigate the effect of small-molecule inhibitors of adhesive interactions under physiologically relevant conditions. Force clamp AFM allows measuring the stabilities of receptor–ligand complexes under sustained discrete tensile loads [64]. A question that this methodology could help address is how adhesive interactions during mating respond to mechanical stresses and whether they are enhanced by them. AFM SCFS protocols exist for the study of interactions between single living mammalian cell pairs [8,43,79]; therefore, it can also be applied to investigate the forces in the adhesive interactions between gametes.

AFM has already proven itself as a singularly powerful tool to investigate single-molecular interactions that govern cellular adhesion. We are confident that the SCFS modality will play a key role in future breakthroughs on cell–cell mating.

## Figures and Tables

**Table 1:** Adhesion systems in mating.

Organism	Receptor	Ligand	References
Mammals	ZP2 ♀ ^1^	Unknown ♂ ^2^	[1,12,14,15,16]
	Izumo1 ♀	Juno ♂	[14,15]
Echinoderms	Unidentified lectins ♂	Sulfated glycans ♀	[17,18,19]
	Bindin ♂	PE ^3^, Ebr1, 350 kDa glycoprotein ♀	[20,21]
*Chlamydomonas*	Sag1 (mt+ cells)	Sad1 (mt- cells)	[22]
Yeasts			
*Saccharomyces cerevisiae*	α-Agglutinin (*MATα* cells)	**a**-Agglutinin (*MAT***a** cells)	[1,25,26,27,28,36] ^5^
Bacteria	MPS ^4^		
*Enterococcus faecalis*	PrgB	e-DNA, lipoteichoic acid	[32,33]

^1^ Ovum; ^2^ spermatozoid; ^3^ phosphatidyl ethanolamine; ^4^ mating pair stabilization; ^5^ reference [36] is the first report of the use of AFM single-cell force spectroscopy to study adhesive interactions in a mating system.

## Data Availability

Not applicable.
